# A Web-Based Intervention to Reduce Decision Conflict Regarding HIV Pre-Exposure Prophylaxis: Protocol for a Clinical Trial

**DOI:** 10.2196/15080

**Published:** 2020-06-15

**Authors:** LaRon E Nelson, Wale Ajiboye, Pascal Djiadeu, Apondi J Odhiambo, Cheryl Pedersen, S Raquel Ramos, Aisha Lofters, Lawrence Mbuagbaw, Geoffrey Williams

**Affiliations:** 1 MAP Center for Urban Health Solution St. Michael's Hospital Toronto, ON Canada; 2 Yale School of Nursing Yale University New Haven, CT United States; 3 Department of Health Research Methods Evidence and Impact McMaster University Hamilton, ON Canada; 4 Dalla Lana School of Public Health University of Toronto Toronto, ON Canada; 5 New York University Rory Meyers College of Nursing New York, NY United States; 6 University of Rochester Rochester, NY United States

**Keywords:** pre-exposure prophylaxis, PrEP, HIV, blacks, prevention, smartphone, mobile phone

## Abstract

**Background:**

HIV pre-exposure prophylaxis (PrEP) is recommended for populations at high ongoing risk for infection. There are noted racial disparities in the incidence of HIV and other sexually transmitted infections (STIs) for African, Caribbean, and Canadian Black (ACB, black) populations in Ontario, Canada. Although blacks represent only 4.7% of the Ontario population, they account for 30% of HIV prevalence and 25% of new infections in the province. The existing clinical public health practice toolkit has not been sufficient to optimize PrEP uptake, despite the overwhelming evidence of PrEP’s efficacy for reducing HIV transmission risk. Since its establishment as an effective HIV prevention tool, the major focus in behavioral research on PrEP has been on understanding and improving adherence. To date, there is no known formalized intervention in place designed to support ACB men and women at high risk of making high-quality decisions regarding the adoption of PrEP as an HIV prevention practice.

**Objective:**

We propose 2 aims to address these gaps in HIV prevention and implementation science. First, the Ottawa Decision Support Framework (ODSF) for use in the PrEP decisional needs of black patients was adapted. Second, the decision support intervention to estimate effect size compared with control conditions in reducing decision conflict and predicting adherence over 60 days was pilot tested.

**Methods:**

In aim 1, we propose a cross-sectional qualitative descriptive study using data collected from key informant interviews with eligible PrEP patients (n=30) and surveys with health professionals (n=20) involved in HIV PrEP management. Data obtained from aim 1 will be used to develop a decision support intervention based on the ODSF. In aim 2, the adopted decision support intervention using a block-randomized design to estimate effect size compared with control conditions in reducing decision conflict and predicting adherence over 60 days was pilot tested. Hypothesis testing will be de-emphasized in favor of generating effect size estimates.

**Results:**

A research award was funded on March 25, 2017 (Multimedia Appendix 1). Ethical approval was received on March 25, 2019 (with supplemental approval received on May 10, 2019). Data collection started on April 9, 2019. As of September 30, 2019, we enrolled 29 patients and 24 health care providers for aim 1. We are currently analysing the data collected for aim 1. Aim 2 is scheduled to start in May 2020.

**Conclusions:**

This study will provide evidence-based information on the decisional needs of black patients who are at risk of HIV and have been offered PrEP. The study will also test the effect of decision support intervention in reducing decision conflict, adoption of PrEP, and adherence to PrEP.

**International Registered Report Identifier (IRRID):**

PRR1-10.2196/15080

## Introduction

### Background

HIV preexposure chemoprophylaxis (PrEP) is recommended for populations at high ongoing risk of HIV infection [1]. There are noted racial disparities in the incidence of HIV and other sexually transmitted infections (STIs) in African, Caribbean, and Canadian Black (ACB) populations in Ontario. Although blacks represent only 4.7% of the Ontario population, they account for 30% of the HIV prevalence and 25% of new infections in the province. There are also especially high rates of HIV in black individuals with a history of STI diagnosis [2,3]. Significant scientific gaps remain regarding the best strategies for supporting PrEP scale-up among blacks. Research evidence on self-determination theory (SDT) indicates that informed and autonomous decision making is a central component in facilitating motivation for long-term maintenance of health behaviors, such as orally taking PrEP daily. This concept has been demonstrated in clinical trials across various populations and health domains [4-9] but has only recently received attention in HIV prevention [10-12]. Considering that decisions regarding whether to start and continue using PrEP can be complex, there are no known studies that have investigated the decisional needs of black patients who are asked to consider taking PrEP. Furthermore, there is no known intervention that provides decisional support to blacks making choices concerning PrEP initiation. There is also a gap in evidence on how the quality of black patients’ decisions to initiate PrEP is related to PrEP adherence.

The existing clinical public health practice toolkit has not been sufficient to optimize PrEP uptake [13-28], despite the overwhelming evidence showing PrEP’s efficacy in reducing HIV transmission risk [29-33]. Since its establishment as an effective HIV prevention tool, the major focus in behavioral research on PrEP has been in understanding and improving adherence [34-40]. To date, there is no known formalized intervention in place designed to support ACB men and women at high risk of making high-quality decisions regarding the adoption of PrEP as an HIV prevention practice. We define *adoption* as an internally endorsed commitment to integrating PrEP into one’s personalized risk reduction plan. This is distinguished from the important concept of *adherence*, which refers to compliance with a medication administration schedule.

### Objectives

We propose 2 aims to address these gaps in HIV prevention and implementation science:

1. Adapting the Ottawa Decision Support Framework (ODSF) for use in the PrEP decisional needs of black patients (Multimedia Appendix 2).
2. Pilot testing the decision support intervention using a 2-arm randomized design to estimate effect size compared with the control condition in reducing decision conflict and predicting adherence over 60 days.

## Methods

### Characteristics of the Research Population

#### Number of Subjects

We will enroll a maximum of 90 (aim 1: n=50, 30 patients, 20 service providers; aim 2: n=40) participants in the study. We expect that 1 in every 3 persons that we screen will enroll in the study; thus, we anticipate screening 140 individuals to reach our enrollment goal.

#### Age of Subjects

We will enroll subjects ≥18 years of age. We excluded children under the age of 18 years because we assessed that the risk of a confidentiality breach was heightened for the youth under the legal age of emancipation. Furthermore, the youth in this age range may require parental/guardian consent for research participation and medical procedures. Complying with such requirements may increase the risk of inadvertent disclosure of information that the youth may have wanted to remain private. With these complexities in mind, we cannot justify the risk of enrolling people aged younger than 18 years as our primary research aim can reasonably be addressed without their inclusion. We are also restricting the enrollment of individuals aged older than 65 years to minimize the potential for confounding due to aging-related neurocognitive factors.

#### Gender of Subjects

We will include both men and women (cisgender and transgender inclusive). We will monitor participant enrollment for aim 1 and aim 2 to ensure that there is an equitable gender distribution in the study. We will not apply gender enrollment targets to the recruitment of health care professionals.

#### Racial and Ethnic Origin

The sample will consist only of individuals who are racially categorized as black. The research questions addressed in this proposal are specific to blacks. The focus on blacks in Canada is due to their disproportionate representation in Canada’s HIV epidemic. Given this, black racial homogeneity is essential to the internal validity of the study, and thus, it is inappropriate to enroll nonblacks into this study. Owing to generations of immigration coming into Canada from countries in Africa and the Caribbean, the black population is ethnically and culturally heterogeneous. We, therefore, expect considerable ethnocultural diversity in the sample; however, we will not set enrollment targets based on culture or ethnicity. We will not apply race/ethnicity criteria to the recruitment of health care professionals.

### Inclusion Criteria

A subject will be eligible for study participation if they (1) are aged at least 18 years, (2) identify as an African, a Caribbean, and/or a Canadian black, (3) currently live in the Greater Toronto Area (GTA), (4) can speak and understand English, and (5) are assessed by the referring health care provider as being a good candidate for starting HIV PrEP.

A health care professional will be eligible for study participation if the following conditions are fulfilled:

1. Are employed at a participating clinical or community-based agency.
2. Have a health care role involved in any component of the PrEP care continuum including the following:
  - Identifying individuals at the highest risk for contracting HIV
  - Increasing HIV risk awareness among these individuals
  - Enhancing PrEP awareness
  - Facilitating PrEP access
  - Linking to PrEP care
  - Prescribing PrEP
  - PrEP clinical management
  - Supporting PrEP adherence
  - Retaining individuals in PrEP care

### Subject identification, Recruitment, and Consent

#### Subject Identification

A venue-based nonprobability sample will be used to identified 12 clinical and community-based agencies. The 12 participating agencies located in downtown Toronto and suburban areas of the GTA with high concentrations of black residents include the following:

- St. Michael’s Family Practice Unit
- Sumac Creek Health Centre
- St. James Town Health Centre
- St. Lawrence Health Centre
- Health Centre at 80 Bond Street
- Health Centre at 410 Sherbourne
- Women’s Health in Women’s Hands Community Health Centre
- Africans in Partnership Against AIDS
- Church Wellesley Health Centre
- Taibu Community Health Centre
- Black Coalition for AIDS Prevention
- Committee for Accessible AIDS Treatment

Staff employed at the participating clinical and community-based sites will identify prospective participants that meet basic study-defined eligibility criteria for HIV PrEP as described in the inclusion and exclusion criteria of this protocol. Once it is determined that a potential participant meets the eligibility criteria, the staff will inform the patient about the study’s existence and inquire if they want to be contacted by a research assistant (RA). If the person agrees to be contacted, then the staff will ask the patient (potential study participant) to provide written consent to release their information to the research team. Once written consent is obtained, the staff will relay the person’s contact information to the study office. An RA will follow up with the patient by phone to further explain the study and to conduct a confirmatory eligibility screen. Eligible participants will be invited (with assistance if necessary) to visit the study office at 209 Victoria Street for informed consent and enrollment.

Some participants identified through the participating clinical and community-based agencies may elect to receive the study’s contact information and call the study office, in lieu of (or in addition to) authorizing site staff to release their personal contact information to the RA. Participants who contact the study office will be given a brief overview of the study and asked if they wanted to learn more details. If the person is interested in knowing more about the study, then the RA will further explain the study and conduct an eligibility screen. Eligible participants will be invited (with assistance if necessary) to visit the study office at 209 Victoria Street for informed consent and enrollment.

#### Subject Recruitment

We will recruit primarily from community-based and clinical providers. Personnel at the selected participating clinical and community-based agencies will assist with recruitment. RAs will be available by phone during the same operating hours as the participating clinical and community-based agencies in order for them to (1) be reached by phone by someone attempting to make a referral and (2) initiate contact with a potential participant within the same business day. The RA’s role in the recruitment process is to screen for initial interest and, if the person is interested, to then screen the potential participant to determine if they are a match to the eligibility criteria. The RA will then initiate informed consent procedures and enrollment into the study.

#### Process of Consent

The RAs will be responsible for obtaining and ensuring informed consent from study participants. The RAs will fully explain the study and answer all questions regarding the participants will be asked to do as part of the study. RAs will receive training on informed consent so that during outreach activities, they can tell potential participants what to expect. In most cases, the RAs will perform the informed consent procedures as a contiguous process with recruitment. We will ensure that all participants know that their participation is completely voluntary and that they can withdraw at any time without repercussions. Once a participant verbally indicates to the RA that all their questions have been satisfactorily answered, we will document that the person has given informed consent to participate by having them sign an informed consent form. The paper copy will be stored in a locked filing cabinet. The informed consent does not end at this phase but continues throughout the entire time that the participant is engaged with the study. In service of this critical point, the RAs are responsible for ensuring that the participant understands what it is that they are being asked to do as part of the study throughout the time that the subject is enrolled, so that their participation always remains informed and volitional.

#### Illiterate and Visually Impaired Participants

Participants who are unable to read in English (defined here as illiterate) or who are visually impaired will have the possibility to select a witness who is literate and has no connection to the research team. The witness should be an adult who can confirm the accurate reading of the consent form to the participant and that the participant has given consent freely. The participant will still sign the informed consent document, and the witness will also sign attesting they understand what the participant is being asked to undertake in the study. Alternatively, participants will be given the option of having a second staff member, (a research staff who does not work on this particular research study or a staff member from the St Michael’s Hospital Family Health Team (SMH FHT) or community agency), witness the consent portion of the interview. Participants can choose the option they prefer.

### Aim 1: Methods and Study Procedures

#### Aim 1: Adapt Ottawa Decision Support Framework for the HIV Pre-Exposure Prophylaxis Decisional Needs of Black Patients

Under this aim, we will investigate 2 research questions: (1) what factors do black patients consider when deciding if to adopt HIV PrEP? and (2) How do SDT constructs of autonomy, competence, and relatedness influence black patients’ decision-making experiences regarding PrEP adoption?

#### Overview and Theoretical Basis of Self-Determination Theory Principles for the HIV Pre-Exposure Prophylaxis Decisional Needs of Black Canadians

SDT is a social psychological theory of motivation that contends that humans are naturally inclined toward health-protecting activities. These natural inclinations are optimized through the support of a human’s basic psychological needs for autonomy (volition and freedom), competence (perceived ability to attain a desired goal), and relatedness (connection to and caring from others). SDT also articulates how sociocultural factors can either facilitate or undermine volition. In this study, we will qualitatively investigate the ways in which autonomy, competence, and relatedness are present in (or absent from) PrEP decision-making experiences of black patients and use this to adapt the ODSF.

#### Evidence for Adaptation of the Ottawa Decision Support Framework for HIV Pre-Exposure Prophylaxis Decisional Needs of Black Patients

Decision support tools are a best evidence strategy in health care and improve the quality of decision making by (1) improving the accuracy of HIV risk assessment, (2) creating realistic outcome probabilities for each decision option, (3) resolving decisional conflict and increasing confidence when choosing among options, and (4) increasing satisfaction with the chosen decision. However, decision support tools have received little attention in HIV prevention and increased uptake of PrEP. Studies show that those at increased risk of HIV seroconversion underestimate their risk of HIV infection and thus may not appreciate the personal relevance of PrEP. For example, in 1 study of 7 public health clinics, 67% of people newly diagnosed with HIV rated their risk for infection as *low* or *no* risk. Studies on PrEP adoption intentions also found that self-assessing one’s behavior as *low risk* was associated with decreased intentions and the likelihood of using among men who have sex with men (MSM). Moreover, using a web-based decision aid is congruent with SDT as it promotes autonomy by eliminating perceived pressure for patients to make immediate decisions during their clinical appointment.

#### Design and Setting

We propose a cross-sectional qualitative descriptive study using data collected from key informant interviews with PrEP eligible patients (n=30) and surveys with health professionals (n=20) involved in HIV PrEP management. The study will take place in the GTA (population. 2.5 million). Over half (59%) of Canada’s black population is settled in the province of Ontario. Moreover, the majority (70%) of black people in Ontario live in GTA, making it the ideal location for this study. The trial procedures will be conducted at sites within the St. Michael’s Hospital (SMH) system, including the SMH Li Ka Shing Knowledge Institute.

#### Procedures

Using the Centers for Disease Control and Prevention guidance for emtricitabine/tenofovir (Truvada) for PrEP and/or the approved generic equivalent, we will identify prospective subjects through SMH FHT sites and community-based agencies. Staff will assess all black patients for PrEP eligibility and interest in initiating PrEP. We will purposively select subjects who want to start PrEP (n=10), do not want to start PrEP (n=10), and remain undecided (n=10) for one-on-one qualitative interviews. We will monitor sexual orientation to ensure MSM are represented in the sample. In the interviews, we will inquire about their (1) concerns about PrEP and barriers to PrEP initiation, (2) normative beliefs about PrEP, and (3) decision-making processes regarding PrEP use. We will also conduct surveys with SMH FHT staff and staff at community-based agencies who assess patient risks and make clinical recommendations for PrEP as well as those that may prescribe and/or support clinical PrEP management. We will use these qualitative findings to guide the adaptation of the ODSF for use in supporting the decisional needs of black patients who are considering PrEP. Patients will receive Can $30 (US $22) for completing the interviews. Providers will receive a Can $40 (US $29) gift card to Amazon on the web for filling in the survey tool as their participation will occur over the course of their work.

#### Data Collection

We will use several sources of data in this study. The data sources are explained as follows.

#### Demographic Surveys

We will administer a brief demographic survey to participants using the Snap Professional software (Snap Surveys). An RA will be present for this administration, although it is possible for the survey to be self-administered if this is what the participant chooses.

#### Semistructured Key Informant Interviews

We will use a semistructured interview guide for participants that includes items designed to address research questions 1 and 2 of aim 1. We will include items that are shown in the literature to impact decision-making quality. For example, we will address aim 1’s question 1 by including items that explore the perceived relevance of PrEP to their clinical situation, decisional conflict, and clarity regarding risk-reduction options and potential outcomes. We will address aim 1’s question 2 by exploring autonomy (eg, Were you told about the choices and effective options that were available to you when you were discussing PrEP?), competence (eg, What are some examples of how you were encouraged to ask questions during the discussion? How did you feel about the responses you received?), and relatedness (eg, Describe how your values were/were not understood during the discussion about PrEP?). The interviewers will also take brief field notes during the interview and will further develop more detailed notes within 24 hours.

#### Structured Surveys

We will administer a structured survey on *Facilitators and Barriers to Decision Support* using the Snap Professional software. An RA will be present to administer the survey, although it is possible for the survey to be self-administered if this is what the health care provider chooses.

#### Ottawa Decision Support Framework Adaptation

We will use inputs from the qualitative findings in aim 1, public health guidelines on PrEP, and the emtricitabine/tenofovir product monograph to tailor the ODSF for use in the Client-Centered Care Coordination (C4) PrEP decision support web-based app.

### Aim 2: Methods and Study Procedures

#### Aim 2: Pilot Test of the Adapted Decision-Support Intervention Using a 2-Arm Randomized Controlled Design

Hypothesis testing will be deemphasized in favor of generating effect size estimates. This aim will investigate 3 research questions. Preliminary hypotheses include the following:

1. H1: PrEP decision support reduces decision conflict in both low decisional conflict (LDC) and high decisional conflict (HDC) groups.
2. H2: LDC + decision support group will be more likely to initiate PrEP than LDC control.
3. H3: LDC PrEP initiators are more likely than HDC PrEP initiators to have serum levels consistent with adherence at 60 days.

#### Randomization

A block randomization strategy will be used to randomize patients in aim 2 into the experimental or control groups. Random blocks of 2, 4, and 6 are used.

#### Pilot Procedures

We will use C4 decision support tool, which is an HTML-5 mobile web-based app that does not require device-specific configurations. Staff will provide participants with a web link to the decision-support app. When possible (ie, when a participant has a smartphone or other applicable device), staff will help participants preprogram the decision support app as both a bookmark and an icon on the participant’s device and the RA will give participants a brief tutorial on its use. Patients assigned to the experimental condition (High Decision Conflict + Decision Support and Low Decision Conflict + Decision Support) will be asked to use the bookmarked link to the decision-support website within the first 14 days (and thereafter as needed) during the study period. The routine care control group will be asked to use a bookmarked link to the frequently asked questions website on emtricitabine/tenofovir for PrEP. All groups will be compared on decision readiness and decision conflict at 14 days, self-reported PrEP initiation at 30 days, and PrEP adherence at 60 days postenrollment.

#### Data Collection

Data on decision conflict and PrEP initiation will be generated by the participant from self-administered assessments via the decision-support web-based app. We will also collect finger-stick blood drops to measure adherence to HIV PrEP at 60-days postenrollment. We will use several sources of data in this study. The data sources are structured survey and blood draw.

#### Patient Surveys

RAs will send participants an email with the link to access the web-based structured survey, which will be programmed in Snap Professional software. All data were entered into Snap Professional software. Once the survey is complete (ie, the submit button is selected), the survey is uploaded automatically to the Snap web host. As soon as this transfer is complete, the survey and its data are automatically removed from the device. We will collect whole blood using a finger-stick procedure and use the microfluid sample for dried blood spot analysis.

#### Measures for Aim 2

In addition to basic demographic data that will be used to describe the sample (eg, age, gender, relationship status), we will use the measures summarized below to assess key variables necessary to address aim 2:

1. The sure test indicates *the probability that a patient experiences clinically significant decisional conflict.*
2. The stage of decision making is a 4-6 item instrument. *Stage of decision making refers to the individual’s readiness to engage in decision making, progress in making a choice, and receptivity to considering or re-considering options.*
3. The Decisional Conflict Scale is a 16-item tool.* The Decisional Conflict Scale (DCS) measures personal perceptions of: (1) uncertainty in choosing options, (2) modifiable factors contributing to uncertainty such as feeling uninformed, unclear about personal values and unsupported in decision making; and (3) effective decision making (in full version) such as feeling the choice is informed, values-based, likely to be implemented and expressing satisfaction with the choice.*
4. The Decision Preparation Scale is a 10-item scale. *The scale assesses a patient’s perception of how useful a decision aid or other decision support intervention is in preparing the respondent to communicate with their practitioner at a consultation visit and making a health decision.*
5. The Health Care Climate Questionnaire (HCCQ) is a 15-item questionnaire. *The HCCQ was designed to be used by patients to report their perceptions of their doctors or their team of health care providers*
6. The PrEP Self-Regulation Questionnaire is a 15-item questionnaire that elicits information regarding why an individual may initiate and/or maintain PrEP usage.
7. The PrEP Use Perceived Competence Scale is a 4-item scale for individuals who elect to use PrEP regarding their perceived confidence to adhere to their decision to use PrEP.

### Payment for Participation

We will implement a modest, modular incentive structure to be applied to participants who enroll and attempt to participate in the study (Table 1). In aim 1, a Can $30 (US $22) cash gratuity will be offered for a 1-hour interview. Health care providers will receive Can $40 (US $29) for the health care provider survey. Participants can receive up to Can $120 (US $86) in aim 2. Participants will be provided with 2 Toronto Transit Commission transit tokens for in-person interviews (enrollment interview and 60-day visit for the finger-stick blood drop). The baseline in-person survey and registration process are expected to take approximately 45 min. The subsequent web-based surveys are expected to take about 15 min; thus, participants in aim 2 are committing to participating for about 1.5 hours of their time. Participants will receive the incentive for enrolling in and completing the study and will also receive it even if they decide (at any point) that they do not wish to continue completing the study activities. If participants know that they will receive the incentive even if they do not complete the procedure (eg, follow-up assessments), then this will reduce the risk that participants are financially coerced to complete any component of the study. These gratuities are also culturally accepted gestures of appreciation for the participants’ generosity in contributing their time and knowledge to the study. Participants will receive incentives immediately after the completion of data collection.

The data collected in this study will not be made available to participants. The data are for exploratory research purposes and are not appropriate for participants to use for medical self-management.

**Table 1:** Participant incentive schedule.

Study procedures		Incentive amount	
**Aim 1 study procedures**			
	Key informant review		Can $30 (US $22)
	Health care provider survey		Can $40 (US $29)
**Aim 2 study procedures**			
	Baseline survey		Can $20 (US $15)
	14-day follow-up		Can $30 (US $22)
	30-day follow-up		Can $30 (US $22)
	60-day follow-up		Can $40 (US $29)

### Subject Withdrawals

We will advise all study participants that their involvement in the study is completely voluntary and that they are free to withdraw at any time, for any reason, without penalty or prejudice. This will be emphasized during the process of obtaining initial informed consent from participants and again during each of the data collection points of the study. Participants who withdraw from the study will be provided with an opportunity to indicate if they also want any data collected up to that point to be used in the study analysis. If a participant does not wish to have their data included in the study analysis, then we will identify the specific data in our database and mark it as *999=not for use in analysis.* We will also keep a record of reasons for participant withdrawals (if the participant wishes to disclose such reasons), to monitor patterns and use these in our research team training sessions to better identify strategies to support retention.

### Risk/Benefit Assessment

#### Risks to Subjects

Participation in the study does not involve more than minimal risk. Potential risks of study participation are outlined as follows.

#### Frustration With Assessments

Potential risks of participation in the study include the frustration that may be encountered in completing assessments, scales, and questionnaires. Subjects are carefully counseled that they may discontinue testing at any time if they find it frustrating or embarrassing. There is also a potential risk of breaching patient confidentiality.

#### Risk for Financial Coercion

We will also implement a modest, modular gratuity structure to be applied to participants who enroll and attempt to participate in the study. A Can $30 (US $22) cash gratuity will be offered for participation in the key informant interview. Health care providers who participate in the structured survey will receive Can $40 (US $29) cash gratuity. Subjects who participated in the pilot trial will receive a modest gratuity at enrollment when they register on the decision aid app via their smartphone device (Can $25 [US $18]), at day 30 when they complete the midpoint survey (Can $30 [US $22]), and at day 60 (Can $45 [US $32]) when they provide a blood sample for adherence assessment. Although the gratuity is very modest, it is possible that some individuals experiencing severe material and financial deprivation may be compelled to participate against their own volition in order to receive the modest gratuities offered.

#### Risks of Unintentional Disclosure of Private Health Information

The greatest potential risk in this study was the potential for a breach of confidentiality which is most likely to occur in situations where the participant has opted to have the RA perform study procedures (eg, study description, informed consent, data collection) outside of one of the participating agencies, such as in their private home. The clinic sites are all equipped with private examination rooms that allow the participant and RA to discuss private health information without the risk of unauthorized persons overhearing protected information. To support participant autonomy and comfort, we will also designate the SMH Li Ka Shing Knowledge Institute as an alternative site for data collection. This site also has private spaces where study procedures can be performed. If a participant desires to complete study procedures in a nonclinic location, the RA cannot guarantee that what is discussed cannot be overheard by others who may be in the immediate vicinity either at a fixed distance away or moving around nearby.

### Legal Implications of Unintentional Disclosure of Private Health Information

It is possible that the participants in this study could be engaged in social and/or legal contractual arrangements with others that may presume mutual monogamy, such as marriage. An unintentional disclosure of sexual behavior information, including same-sex behavior, could provide material evidence for litigation against the study participant by an allegedly injured party. This could have both legal and financial consequences for the study participants. This risk is more likely to occur during a visit outside of the clinical setting where others who are not bound by privacy regulations may be in close enough proximity to hear clinical discussions between the participant and the RA.

### Protections Against Risk and Minimizing Risk of Unintentional Disclosure of Private Health Information

#### Emphasis on Voluntary Nature of the Study

We will emphasize to all study participants that their involvement in the study is completely voluntary and that they are free to withdraw at any time, for any reason, without penalty. This will be emphasized during the process of obtaining initial informed consent from participants and again before enrollment and data collection. Participants who withdraw from the study will be provided with an opportunity to indicate whether they also want any data collected up to that point to be used in the study analysis. We will also keep a record of reasons for participant withdrawals (if the participant wishes to disclose such reasons), such that we can try to monitor patterns and use these in our research team training sessions to better identify strategies to support study retention.

#### Use of Emergency Protocols for Patients Experiencing Psychological Distress

During the course of the study, it may become apparent that a participant is experiencing psychological distress of some sort that warrants further clinical assessment and possibly intervention. This distress could be experienced at any time during the course of the study. However, there is very little evidence to support that behavioral and psychosocial surveys trigger acute episodes of psychological distress for study participants in behavioral research studies; nonetheless, it is remotely possible. If a participant appears to be in psychological distress during the interview, the RA will bring the interview to a close. After that, they will sensitively engage the individual to determine if they wish to talk about what causes them distress. The RA will also ask questions to ascertain if the participant is receiving any support for their distress (eg, friends, family, or professionals), and if not, whether they feel they would benefit from support. If they wish to receive support, the RA will provide them with a list of professionals to talk to.

It is more likely that a participant may experience distress upon receiving an HIV diagnosis. After initiating contact with the RA, participants will be provided with the Canadian AIDS Treatment Information Exchange (CATIE)’s contact information, including their website. CATIE is a nationally respected Non Governmental Organization⸺funded federally and provincially⸺that provides evidence-informed, sex-positive, plain language HIV and Hepatitis C resources in French and English, in print, via phone, and through collect calls. We do not know how many (if any) participants will HIV seroconvert during the course of their participation in the study. All participating clinics have many years of experience in planning for and responding to situations in which patients are unable to manage their psychological distress and require support from clinical staff. We will use clinical site-specific security and emergency protocols to handle potential situations that arise at the site.

#### Reminder About Risks of Disclosures Outside of Clinic Environments

If a participant wishes to provide informed consent and survey procedures outside of the SMH FHT’s, SMH Li Ka Shing Knowledge Institute, or community agency study sites, we will remind them of the limitations this poses for maintaining confidentiality. We will further inform the participant that even if no one is physically present at the location they choose, we cannot guarantee the degree to which the discussion of any clinical information is *private*. We will remind participants that they are not compelled to share any information about which they do not feel comfortable. We will also advise participants of the advantages of study procedures to take place in the preapproved study sites. We want participants to be forthcoming with their information; however, they will be reminded that they are not compelled to share any information that they feel may jeopardize their health or social status.

#### Benefits to Subjects

##### Direct Benefits to Participants

There are no direct benefits to the participants for enrolling in this research study.

##### Benefits to Others

This study has important potential benefits to the community and to primary HIV prevention science. The study will generate knowledge that will be used to inform the development of a decision support intervention to help participants make more informed choices about their engagement in clinical HIV prevention. Furthermore, this proposed study will form a foundation to support more studies of program and implementation science that aims to make scientific advances through research while at the same time making gains and improvements in health outcomes in real-world practice environments.

#### Important Knowledge to Be Gained

Results from this pilot will generate data for the preliminary studies section of a research grant application. The results will also help us determine how providing decision-support and improving decision quality can enhance local health department efforts to link and support black patients’ maintenance of HIV PrEP.

#### Alternatives to Participation

In this study, participants chose to withdraw their participation at any time. To our knowledge, there are no alternatives for PrEP-related decision support available in the GTA. There are several clinic-based and web-based resources that provide information regarding HIV PrEP. Participants can make use of any available sources of PrEP information via the agency from which they were referred. Any participant who wishes to discontinue participation in the study will be offered a leaflet that lists HIV PrEP informational resources.

#### Confidentiality of Data and Information Storage

We will only collect the minimum contact information necessary to be able to reach participants for scheduling and reminders about the study visits and data collection. We will collect a participant’s first name (or alias), cell phone number, and email addresses. We will not collect information such as home address and work phone number so as to avoid the risk that unauthorized persons can apprehend and use it to identify study participants. We will also ask participants to provide the number of 1-2 trusted family members or friends that we can call in the event of a medical or legal emergency, or if we are unable to reach the participant for a follow-up. We will not provide family or friend contacts with any information about the study to maintain participant confidentiality.

The study team will use electronics-based surveys using the Snap Professional software for data collection. Please note that the Snap Professional Software has been reviewed and approved for use by St. Michael Hospital’s: Peter Lambert, Manager of Privacy and Security and Rino La Grassa, Research Applications Support Specialist (Information Communication Technology). The Snap server utilized is owned by the Centre for Urban Health Solution Survey Research Unit and is located inside the SMH network.

No identifiable participant information or de-identified participant information will be stored locally. The RA will not record any client contact information in the Snap Professional software. Original lab reports (source documentation) will be stored onsite at the SMH lab and maintained by the site principal investigator (LN) at SMH when the study concludes.

All survey data were entered into Snap Professional software. Once the survey is complete (ie, the submit button is selected), the survey is uploaded automatically to the Snap web host. Before it reaches the web host, it must first travel through a secure socket layer (SSL), which is where the data is encrypted. Any partially completed surveys will undergo the same process and can be retrieved from the web host to complete at a later date. As soon as this transfer is complete, the survey and its data are automatically removed from the device. Data stored on the secure web host server will be deleted after study completion. Before deletion, the research team will confirm that all completed surveys have been downloaded to the SMH secure server. Once confirmed, the surveys will be deleted from the Snap webhost, and this will be documented in an Excel file. Again, both the Snap web host and SSL tunnel have undergone a TRA to ensure that the data transferred and stored remains encrypted and secure.

No identifying information (eg, name, address, phone number, place of employment) was collected in the behavioral survey. The behavioral survey data is maintained separately from the data file, which includes participants’ contact information to reduce the risk that survey responses are linked to participant identities. No identifying information is listed on the blood samples of the dried blood spots. The dried blood spot transport media will be labeled only with participant identification codes. ID codes will be used to match the emtricitabine/tenofovir concentration with the self-report data variables for each participant.

#### Research Information in Medical Records

No information generated during the study will become part of the participant’s medical record, unless requested by the participant. If such a request is posed by the participant, they will be advised of the potential risks and benefits to doing so. The request will be honored, provided it does not transgress the privacy and confidentiality requirements of Ontario.

No one outside of the principal investigator, RA, research manager, study medical director, and a medical provider with a clinical *need to know* will have access to any identifiable data. The RA will need access to basic identifying data such as name and phone number for follow-up contact purposes to aid in recruitment, enrollment, and data collection. If an urgent clinical issue arises (such as a participant experiencing acute psychological distress), it may be necessary to link a participant’s contact information to the subject’s identity in order to provide clinical follow-up. No data will be shared without the participant’s written informed consent to release medical information. It is in keeping with the principle of beneficence to ensure continuity of care by making it possible (not mandatory) for participants to share information with the medical provider.

#### Data Analysis and Data Monitoring

##### Planned Statistical Analysis

###### Aim 1: Analysis Plan

Interview data will be transcribed verbatim and subjected to qualitative content analysis. After uploading the transcripts into NVivo, we will read each transcript and use *in vivo* (open) coding function to bracket text segments that describe factors that influence decision-making regarding PrEP adoption. We will also develop *a priori* codes based on SDT constructs and use these, along with open codes, to investigate research questions. We will use data display tables to arrange codes by decision-group (adopted vs declined vs undecided) and examine the text associated with each code, consolidating overlapping codes and clustering codes that fit together. The code clusters will be reviewed within and across decision groups to interpret and describe how they address our research questions. We have successfully used these analytic strategies in previous qualitative research.

###### Aim 2: Analysis Plan

To compare conditions, we will use analysis of covariance procedures. When we compare the 4 conditions, our primary interest will be the tests of participants who received the decision-support intervention versus the information-only control. We will test whether the decision quality, PrEP initiation, and PrEP adherence differ between conditions controlling for baseline decision-quality score. We did not conduct a power analysis to calculate sample size because we de-emphasized hypothesis testing in favor of generating effect size estimates to inform the development of a larger study.

###### Data Handling

The statistical analyses for the study will be performed at McMaster University by LM. Once all self-report surveys and biological data have been entered into Snap Professional software, LN will download a complete database with no identifying information and provide it to LM for analysis. No identifiable information was included in the downloaded database.

###### Data and Safety Monitoring

LN and Dr Jesleen Rana will review patient data biweekly, commencing 14 days after the first participant is enrolled in the trial of the decision support intervention. Dr Rana will chair the patient safety review team. The research manager will prepare routine safety data reports for review by the research team. The study team will meet monthly or as needed throughout the study implementation to review safety data as well as discuss and address any potential safety concerns. The study team will agree on the content and format of safety data reports before study implementation. Furthermore, as this study does not have data safety monitoring board oversight, the SMH Institutional Review Board may also review aggregate⸺or individual level−safety data.

The data and safety monitoring responsibilities of the study team will include the following:

1. Maintain confidentiality of the data and results of the monitoring.
2. Review the research protocol and plans for data safety and monitoring.
3. Review monthly (or more frequently as needed).
4. Participant recruitment:
  - Retention between recruitment and data collection
  - Participant risk-benefit ratio
  - Unanticipated adverse effects
5. Monitor reports of related studies to determine if this study needs to be changed or terminated.
6. Review proposed modifications to the study before implementation.
7. Determine if the study should continue as designed, modified, or terminated based on the data available at the review meeting.

In addition to the routine safety data reviews, the study team will convene on an ad hoc basis to make decisions regarding the handling of any significant safety concerns. If necessary, experts external to the study representing expertise in the fields of community-based research, biostatistics, or medical ethics may be invited to join the safety review. A recommendation to stop the study may be made by the study team at any such time that the team agrees an unacceptable type and/or frequency of adverse events has been observed. In the unlikely event that the study team has serious safety concerns that lead to a decision to permanently discontinue the study for all participants and stop accrual into the study, the principal investigator will immediately notify the SMH Institutional Review Board.

## Results

A research award was funded on March 2017. Ethical approval was received on March 25, 2019 (with supplemental approval received on May 10, 2019). Data collection started on April 9, 2019. As of September 30, 2019, we enrolled 29 patients and 24 health care providers for aim 1. We are currently analyzing the data collected for aim 1. Aim 2 is scheduled to start in May 2020.

## Discussion

This study will provide evidence-based information on the decisional needs of black patients who are at risk of HIV and have been offered PrEP. The study will also test the effect of the decision support intervention in reducing decisional conflict, adoption of PrEP, and adherence to PrEP.

## Appendix

Multimedia Appendix 1 SON peer review letter.

Multimedia Appendix 2 Ottawa Decision Support Framework (ODSF).

## Abbreviations

ACB: African, Caribbean, and Canadian Black
C4: Client-Centered Care Coordination
CATIE: Canadian AIDS Treatment Information Exchange
GTA: Greater Toronto Area
HCCQ: Health Care Climate Questionnaire
HDC: high decisional conflict
LDC: low decisional conflict
MSM: men who have sex with men
ODSF: Ottawa Decision Support Framework
PrEP: pre-exposure prophylaxis
RA: research assistant
SDT: self-determination theory
SMH FHT: St Michael’s Hospital Family Health Team
SSL: secure socket layer
STI: sexually transmitted infection
