# Supplementary material for: A Web-Based Intervention to Reduce Decision Conflict Regarding HIV Pre-Exposure Prophylaxis: Protocol for a Clinical Trial
Source: JMIR Res Protoc. 2020 Jun 15;9(6):e15080. doi: 10.2196/15080 (PMC7324994; doi:10.2196/15080)
Supplement: Multimedia Appendix 1 [file resprot_v9i6e15080_app1.pdf]

SON SMD Pilot Grant Review Form

<u>Principal Investigator</u>: LaRon Nelson (SON) and Geoff Williams (General Medicine, Psychiatry)
($50,000)

<u>Proposal Title:</u> "Pilot Randomized Controlled Trial of a Decision Support Intervention Adapted for HIV Pre-Exposure Prophylaxis Adoption and Adherence among African, Caribbean and Black Canadian STD Clinic Patients" (1/1/17-12/31/17)

<u>NIH High Priority Area:</u> Research to Reduce Racial/Ethnic Disparities in HIV Infections

<u>Strengths</u>: Important knowledge gap to fill in a unique high-risk population. Strong study design. Establishes a track-record for CFAR research in Toronto, Canada.

<u>Weaknesses</u>: Lack of defined role for SMD PI (G Williams), so not directly responsive to the CFAR goal of meaningfully engaging both PIs in the HIV research field.

<u>Rank Order (1 = highest preference and should be funded first and 3 = lowest preference for funding)</u>

(consider resubmission for a general CFAR pilot)

SON SMD Pilot Grant Review Form

Principal Investigator: LaRon Nelson (SON) and Geoffrey Williams (SMD/Medicine) $50,000)
Proposal Title: Pilot Randomized Controlled Trial of Decision Support Intervention Adapted for HIV Pre-Exposure Prophylaxis Adoption and Adherence among African. Canadian, and Black Canadian STD Clinic Patients.
NIH High Priority Area: (several NIH high-priority topics) namely, reducing incidence of HIV/AIDS (specifically, methods of PrEP delivery that mitigate adherence issues), and reducing health disparities.

Strengths
-Taking PrEP represents an important decision for at-risk ABC patients. Using the Ottawa Decision Aid Framework (ODAF) to optimize patient informed decision-making represents an important aim that could improve the quality of decision making for this population.
-Integrating Self Determination Theory (SDT) into this framework provides a strong theoretical foundation that can potentially inform best steps for operationalizing a decision aid (DA).
-Potentially, application of ODAF and SDT to qualitative findings from the relevant population could produce a novel, scalable DA that improved informed decision-making surrounding PrEP for the ABC population.
-The investigative team is well qualified. Dr. Nelson has extensive experience working with the HIV and HIV at-risk population including the ABC MSM population. Dr. Williams is an expert in application of SDT to health care.
-The environments of the SON, SMD/Medicine, and UR CFAR are supportive.
-Conducting qualitative interviews with users and with clinical providers is consistent with best practices, i.e. with the International Patient Decision Aid Standards IPDA).

Weaknesses
-Details are lacking on exactly how SDT will guide design of the DA. How might SDT result in a design that is different than if the team simply followed IPDA guidance? There is no mention of apha, i.e. usability and comprehension recommended by the IPDA or beta testing prior to piloting it in an RCT. This testing helps ensure that the DA will be used by participants as intended; that the core issues are being understood as intended; that feasibility issues have been resolved; and that SDT is embedded into it.
-The use of a RCT to determine effect size seems premature at this juncture given the absence of alpha and beta testing.
-It is not clear what will be learned by randomizing participants to a control group. It is not clear what outcome is being measured? Is it uptake of PrEP, perceived patient autonomy in decision-making, decision conflict or some other construct?
-It is not clear what measures will be used to assess the RCT in aim 2 and what results will indicate whether or not this DA warrants further evaluation in a full scale RCT.

Rank Order (1 = highest preference and should be funded first and 3 = lowest preference for funding) and rationale for rank

A DA for PrEP for ABC could significantly improve informed decision-making for the ABC population. However, lack of planned alpha and beta testing prior to the trial may hinder feasibility, acceptability, and usability.

SON SMD Pilot Grant Review Form

Principal Investigator: LaRon Nelson (SON) and Geoffrey Williams (Psychiatry) ($50,000)

Proposal Title: "Pilot Randomized Controlled Trial of a Decision Support Intervention Adapted for HIV Pre-Exposure Prophylaxis Adoption and Adherence among African, Caribbean and Black Canadian STD Clinic Patients" (1/1/17-12/31/17)

NIH High Priority Area: Research to Reduce Racial/Ethnic Disparities in HIV Infections

Strengths

- **PrEP implementation in minority MSM has been difficult so real need for some targeted interventions.**
- **Researchers bring complimentary skills. Dr. Nelson seems to have extensive experience with target population and with HIV prevention interventions.**
- **Very well written – clear plan to move forward with subsequent funding**

Weaknesses

- **It seems there are 4 sites with 2 RAs in Canada so may be hard to enroll subjects in timely fashion in order to get to the larger analysis in the second part of the project**
- **Could consider changing adherence to 90 days (instead of 60 or in addition to 60) to match usual PrEP follow up protocols**

Rank Order (1 – highest preference and should be funded first and 3 being lowest preference for funding) and rationale for rank

Well written project, attempting to address PrEP uptake in high risk minority MSM. I thought my Number 1 ranking more likely to lead to future funding.
