# Supplementary material for: A Web-Based Intervention to Reduce Decision Conflict Regarding HIV Pre-Exposure Prophylaxis: Protocol for a Clinical Trial
Source: JMIR Res Protoc. 2020 Jun 15;9(6):e15080. doi: 10.2196/15080 (PMC7324994; doi:10.2196/15080)
Supplement: Multimedia Appendix 2 [file resprot_v9i6e15080_app2.pdf]

Ottawa Decision Support Framework to Address Decisional Conflict

The Ottawa Decision Support Framework (Fig 1) uses concepts and theories from general psychology (Tversky & Kahneman, 1981), social psychology (Ajzen & Fishbein, 1980), decision analysis (Keeney, 1982), decisional conflict (Janis & Mann, 1977), values (Fischoff, Slovic & Lichtenstein), social support (Norbeck, 1988; Orem, 1995), and self efficacy (Bandura, 1982).

Figure 1. Ottawa Decision Support Framework

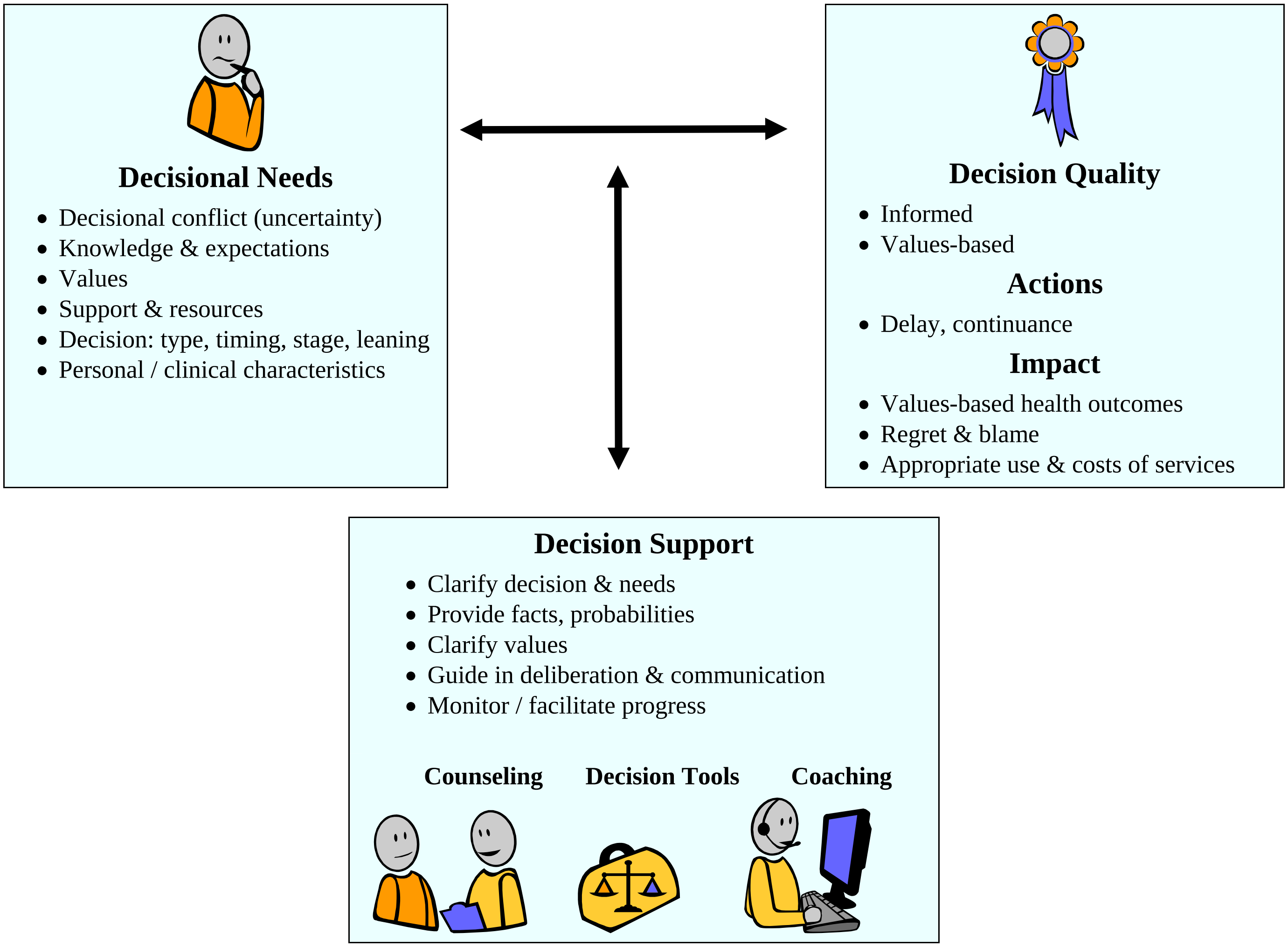

The framework applies to all participants involved in decision making, including the individual, couple, or family, and their health practitioner. The framework asserts that participants' decisional needs will affect decision quality (informed, values-based choices), which in turn affects actions or behaviour (e.g. delay), health outcomes, emotions (regret, blame), and appropriate use of health services. (See **Glossary of Terms for Ottawa Decision Support Framework**)

Decision support in the form of clinical counselling, decision aids and coaching can improve decision quality, by addressing unresolved decisional needs.

AM O'Connor, Ottawa Decision Support Framework to Address Decisional Conflict. © 2006.
Available from www.ohri.ca/decisionaid.

References

1. Orem DE. Nursing: Concepts of practice. 5th ed. Toronto: Mosby; 1995.
2. Keeney, RL. Decision analysis: An overview. Operations Research 1982; 30:803-38.
3. Janis IL, Mann L. Decision making. New York: The Free Press; 1977.
4. Tversky A, and Kahneman D. The framing of decisions and the psychology of choice. Science 1981; 211:453-58.
5. Fischhoff B, Slovic P, Lichtenstein S. Knowing what you want: measuring labile values. In: Wallsten TS, editors. Cognitive processes in choice and decision behavior. Hillsdale (NJ): Lawrence Erlbaum Associates Inc.; 1980.
6. Norbeck JS. (1988). Social Support. Annual Rev. Nur. Res. 6:85-109.
7. Ajzen I, and Fishbein M. Understanding attitudes and predicting social behaviour. Englewood Cliffs: Prentice Hall; 1980.
8. Bandura A. Self-efficacy mechanism in human agency. American Psychologist 1982;37: 122-147.

Glossary of Terms for the Ottawa Decision Support Framework

DECISIONAL NEEDS

DECISION

Type: class or characteristic of the choice that needs to be made [e.g. developmental transition or clinical options (screen, test, treat, palliate]; number of options, degree of risk/uncertainty, seriousness of outcomes, whether it is irrevocable
Timing: time frame or urgency with which a decision needs to be made
Stage: phase of decision making: not thinking about options; considering options; close to selecting an option; taking steps towards implementing option; have already carried out choice. Categories are similar to Prochaska's Stages of Change (1), with one important difference. Deciding not to change is a viable option because often there is no recommended course of action, e.g. amniocentesis.
Leaning: inclination to choose one option over the other

DECISIONAL CONFLICT

uncertainty about course of action to take when choice among options involves risk, loss, regret, challenge to personal life values

KNOWLEDGE & EXPECTATIONS

Knowledge: cognizance of the health problem or situation, options, and outcomes
Expectation: perceived likelihood or probability of outcomes of each option

VALUES

desirability or personal importance of outcomes of options

SUPPORT & RESOURCES

Others' opinions/ practices: perceptions of what others decide or what others think is the appropriate choice. This may include a person's spouse, family, peers, and practitioner(s). For practitioners: the patient, professional peers, and personal network
Pressure: perception of persuasion, influence, coercion from important others to select one option
Role in decision making: the way a participant is or wants to be involved in decision making; do they prefer to: make the choice themselves after considering opinions; share decision making with another; have others decide after considering their opinion
Experience: past exposure to the situation, options, outcomes, decision making process
Self-efficacy: confidence or belief in one's abilities in decision making, including shared decision making
Motivation: readiness and interest in decision making, including shared decision making
Skill: abilities in making and implementing a decision
External support: Available, accessible assets from others that are required to make and implement the decision. Types include: information, advice, emotional support, instrumental help, financial assistance, health & social services. Sources include: social networks, professional networks, support groups, voluntary agencies, and the formal health care, education, and social sectors

PERSONAL & CLINICAL CHARACTERISTICS

Patient: Age, gender, education, marital status, ethnicity, occupation, locale, diagnosis & duration of condition, health status (physical, emotional, cognitive, social)
Practitioner: age, gender, ethnicity, clinical education, specialty, practice locale, experience, counselling style

DECISION SUPPORT

PATIENT DECISION AIDS

Evidence-based tools to prepare people to participate in making specific and deliberated choices among healthcare options in ways they prefer. They supplement (not replace) clinician's counseling and aid decision making by: a) providing evidence-based information about a health condition, the options, associated benefits, harms, probabilities, and scientific uncertainties; b) helping people to recognize the values-sensitive nature of the decision and to clarify the value they place on the benefits, harms, and scientific uncertainties. Strategies include: describing the options in enough detail that clients can imagine what it is like to experience the physical, emotional, and social effects; and guiding clients to consider which benefits and harms are most important to them; and c) providing structured guidance in the steps of decision making and communication of their informed values with others involved in the decision (e.g. clinician, family, friends).

DECISION COACHING

Support provided to people facing a decision by a trained facilitator who is supportive but neutral in the decision. Coaching can be provided face to face (individual, group) or using communication technologies (telephone, Internet). Decision coaching is used alone or in combination with patient decision aids. The strategies may include: a) clarifying decision and monitoring needs; b) facilitating access to evidence-based information, verifying understanding, clarifying values, building skills in deliberation, communication, and accessing support; and c) monitoring and facilitating progress in decision making and decision quality.

DECISION QUALITY

QUALITY OF THE DECISION

The extent to which the chosen option best matches informed clients' values for benefits, harms, and scientific uncertainties

QUALITY OF THE PROSESS OF DECISION MAKING

The extent to which a person is helped to: a) recognize that a decision needs to be made; b)know about the available options and associated procedures, benefits, harms, probabilities, and scientific uncertainties; c) understand that values affect the decision; d) be clear about which features of the options matter most to them (e.g. benefits, harms, and scientific uncertainties); e) discuss values with their clinician(s); and f) become involved in decision making in ways they prefer.

AM O'Connor, Ottawa Decision Support Framework to Address Decisional Conflict. © 2006.
Available from www.ohri.ca/decisionaid.
